# Supplementary material for: Effects of emerging SARS-CoV-2 on total and cause-specific maternal mortality: A natural experiment in Chile during the peak of the outbreak, 2020–2021
Source: PLOS Glob Public Health. 2024 Jul 11;4(7):e0002882. doi: 10.1371/journal.pgph.0002882 (PMC11238951; doi:10.1371/journal.pgph.0002882)
Supplement: S1 Table — (DOCX) [file pgph.0002882.s001.docx]

| Table S1. Characteristics of the ARIMA models, selection criteria and accuracy indicators of the prediction of the maternal mortality ratio (MMR) groups of mortality, in Chile (1997-2021) | | | | | | | |
| --- | --- | --- | --- | --- | --- | --- | --- |
|  | (p, d, q) | AIC | BIC | Log | RMSE | MAE | Accuracy (%) |
| MMR Total | (2,2,0) | 117.72 | 120.85 | -55.86 | 3.15 | 2.44 | 87.69 |
|  |  |  |  |  |  |  |  |
| **RMM by groups of causes** | | |  |  |  |  |  |
|  | |  |  |  |  |  |  |
| Direct causes | (1,2,1) | 94.64 | 97.78 | -44.32 | 1.79 | 1.30 | 79.67 |
|  |  |  |  |  |  |  |  |
|  |  |  |  |  |  |  |  |
| Indirect causes | (4,2,0) | 94.38 | 99.6 | -42.19 | 1.57 | 1.16 | 72.73 |
|  |  |  |  |  |  |  |  |
| Indirect respiratory causes | (3,1,0) | 113.63 | 118 | -52.82 | 2.46 | 1.84 | - |
|  |  |  |  |  |  |  |  |
|  |  |  |  |  |  |  |  |
| Indirect no respiratory causes | (2,2,1) | 93.22 | 97.4 | -42.61 | 1.61 | 1.27 | 65.37 |
|  |  |  |  |  |  |  |  |
|  |  |  |  |  |  |  |  |
| Indirect infectious causes | (1,1,0) | 136.05 | 138.23 | -66.03 | 4.68 | 3.22 | - |
|  |  |  |  |  |  |  |  |
|  |  |  |  |  |  |  |  |
| Indirect non-infectious causes | (0,2,2) | 87.96 | 91.09 | -40.98 | 1.51 | 1.12 | 64.84 |
|  |  |  |  |  |  |  |  |
| (p, d, q): components of the model where p represents the largest number of lags of the autoregressive parameter, d is the degree of differentiation of the series, and q is the largest number of components of the moving average; AIC: Akaike information criterion; BIC: Bayesian information criterion; Log: log likelihood; RMSE: Root-Mean-Square Error; MAE: mean absolute error; Accuracy (%) percentage accuracy of the prediction. | | | | | | | |
