# Supplementary material for: Effects of emerging SARS-CoV-2 on total and cause-specific maternal mortality: A natural experiment in Chile during the peak of the outbreak, 2020–2021
Source: PLOS Glob Public Health. 2024 Jul 11;4(7):e0002882. doi: 10.1371/journal.pgph.0002882 (PMC11238951; doi:10.1371/journal.pgph.0002882)
Supplement: S1 File — (DOCX) [file pgph.0002882.s003.docx]

Resumen

Este estudio estimó los efectos de la pandemia de COVID-19 en la mortalidad materna en Chile entre 2020 y 2021. Se realizó un experimento natural utilizando datos oficiales sobre muertes maternas y nacidos vivos (NV) entre 1997 y 2021. Los efectos del brote de SARS-CoV-2 se evaluaron utilizando series de tiempo interrumpida (STI) y un modelo autorregresivo integrado de media móvil (ARIMA) para pronosticar los valores esperados de las RMM e intervalos de confianza del 95% (IC del 95%). En Chile, siguiendo las sugerencias de la OMS, las muertes maternas agravadas por el SARS-CoV-2 se asignan al código O98.5 (infeccioso indirecto no respiratorio) acompañado por el código U07.1 o U07.2, dependiendo de la confirmación de la presencia o ausencia del virus. El análisis de STI reveló que el brote de SARS-CoV-2 afectó la RMM debido a causas indirectas, con un mayor aumento de las causas indirectas no respiratorias que de las respiratorias. El pronóstico ARIMA fue consistente con la STI, mostrando que la RMM esperada por causas indirectas fue de 3.44 (IC 95%: -0.13-7.01) en 2020 y de 1.55 (IC 95%: -3.24- 6.34) en 2021, sustancialmente más bajas que las razones observadas (9.65 y 7.46/100,000 NV, respectivamente). Para causas no respiratorias, los valores observados de la RMM para 2020 (8.77/100,000 NV) y 2021 (7.46/100,000 NV) duplicaron la predicción de 4.02 (IC 95%: 0.44-7.61) y 3.83 (IC 95%: -0.12-7.79), respectivamente. No se encontró ningún efecto significativo sobre las muertes obstétricas directas. Durante 2020-2021, se observó un aumento en la RMM en Chile atribuible al SARS-CoV-2. La pandemia contribuyó a un incremento en la RMM por causas indirectas, especialmente aquellas no respiratorias e infecciosas. Por otro lado, la RMM por causas obstétricas directas se vio menos afectada. Esto sugiere que la pandemia tuvo un impacto desproporcionado en la salud materna al agravar condiciones no relacionadas con el embarazo, el parto o el puerperio, más que aquellas directamente vinculadas a complicaciones obstétricas.
