# Supplementary material for: Effects of emerging SARS-CoV-2 on total and cause-specific maternal mortality: A natural experiment in Chile during the peak of the outbreak, 2020–2021
Source: PLOS Glob Public Health. 2024 Jul 11;4(7):e0002882. doi: 10.1371/journal.pgph.0002882 (PMC11238951; doi:10.1371/journal.pgph.0002882)
Supplement: Attachment — Submitted filename: Response to Reviewers.docx [file pgph.0002882.s004.docx]

May 9, 2024

Dear Editors

PLOS Global Public Health

Response to reviewers’ comments

First and foremost, we express our gratitude to the reviewers and editors for the constructive feedback, which will contribute significantly to enhancing the quality of our manuscript. This correspondence serves as a resubmission of our manuscript titled "Effects of emerging SARS-CoV-2 on total and cause-specific maternal mortality: A natural experiment in Chile during the peak of the outbreak, 2020-2021"

Kindly refer to the responses provided in the table below:

| **№** |  | **Comments** | **Response to reviewers** |
| --- | --- | --- | --- |
| 1 | editor | 1. Please amend the title to remove "Title" | The title was amended (see page 1, line 1) |
|  |  |  |  |
| 2 | editor | 2. We do not publish any copyright or trademark symbols that usually accompany proprietary names, eg  ©, ®, ™  (e.g. next to drug or reagent names). Please remove all instances of trademark/copyright symbols throughout the text, including  ® on page 7. | The trademark symbols were removed (see page 3, line 205) |
|  |  |  |  |
| 3 | editor | [3. In the online submission form, you indicated that "The data underlying this article is available from Departamento de Estadísticas de Información de Salud del Gobierno de Chile, https://deis.minsal.cl/. The derived data generated in this research will be shared on reasonable request to the corresponding author". All PLOS journals now require all data underlying the findings described in their manuscript to be freely available to other researchers, either 1. In a public repository, 2. Within the manuscript itself, or 3. Uploaded as supplementary information.](https://deis.minsal.cl/) |  |
|  |  | This policy applies to all data except where public deposition would breach compliance with the protocol approved by your research ethics board. If your data cannot be made publicly available for ethical or legal reasons (e.g., public availability would compromise patient privacy), please explain your reasons by return email and your exemption request will be escalated to the editor for approval. Your exemption request will be handled independently and will not hold up the peer review process, but will need to be resolved should your manuscript be accepted for publication. One of the Editorial team will then be in touch if there are any issues. | Agreed, the database was uploaded as supplementary material in a new document. In the Data Sharing Statement, we modified it to indicate that the data underlying this article were accessed from the Departamento de Estadísticas de Información de Salud del Gobierno de Chile (https://deis.minsal.cl/). The derived data generated in this research is available in the supplementary material  (see page 13, lines 487-488). |
|  |  |  |  |
| 4 | Reviewer 1 | • Standardization of Terminologies: Throughout the manuscript, the authors use terms such as ‘SARS-CoV-2’, ‘emerging SARS-CoV-2’, ‘SARS-CoV-2 pandemic’, and ‘SARS-CoV-2 outbreak’. In some paragraphs, it is unclear whether the authors are specifically referring to cases of infection by the SARS-CoV-2 virus in pregnant and postpartum women, or to the scenario of the COVID-19 pandemic (which involved a set of factors that may have influenced maternal mortality, e.g., infections by SARS-CoV-2 virus, potential restrictions on access to healthcare services, disruption of prenatal consultation routines; inequalities in access to vaccination; resources and health policies employed in addressing the health crisis, among others). Therefore, I suggest that the authors standardize the terminologies, using SARS-CoV-2 to refer to the virus, and COVID-19 pandemic to refer to the period of the Public Health Emergency of International Concern due to SARS-CoV-2. | Thanks for the recommendation. Terminologies were standardized as per the reviewer's recommendation throughout the manuscript. |
|  |  |  |  |
| 5 | Reviewer 1 | • Title: I also recommend including the period in the title: “Impact of (…) in Chile during the peak of the SARS-CoV-2 outbreak, 2020-2021” | The period was included in the title (see page 1, line 3). |
|  |  |  |  |
| 6 | Reviewer 1 | • Abstract: For better alignment with the objective described in the main text of the manuscript, I suggest describing the background as “This study estimated the effects of the COVID-19 pandemic on maternal mortality in Chile between 2020 and 2021.” | The previous phrase was replaced by the one recommended by the reviewer (see page 2, lines 44-45). |
|  |  |  |  |
| 7 | Reviewer 1 | • Introduction: In the last paragraph of introduction, it is suggested to omit the sentence "To achieve this, we employed a time-series design exploiting long-term annual trend information and used ARIMA models to forecast expected mortality under the hypothesis that previous mortality trends would continue in the absence of the pandemic virus-related mortality burden". These details should be allocated in the methods section. | The sentence at the end of the paragraph was removed, as per the reviewer's recommendation (see page 1, lines 108-111). |
|  |  |  |  |
| 8 | Reviewer 1 | • Methods: a) Please, present a descriptive table showing the number and percentage of maternal deaths in Chile, according to the groups of causes and the year of occurrence. If possible, also specify the number and percentage of maternal deaths due to COVID-19 in 2020 and 2021. This would be useful for viewing the distribution of these deaths before and during the COVID-19 pandemic. | Thanks for the recommendation. Nevertheless, the maternal mortality rate (MMR) was preferred over the maternal mortality percentage because it provides a more precise and comparable measure of maternal mortality in different populations or regions. In this sense, the database containing cases of maternal death and Maternal Mortality Ratio (MMR) is provided in the supplementary material. Specifying cases of maternal death attributed to SARS-CoV-2 is challenging because the O98.5 code encompasses other viral diseases that complicate pregnancy, childbirth, and the puerperium. |
|  |  |  |  |
| 9 | Reviewer 1 | b) Did the authors work with the underlying cause of maternal death? The authors need to specify the selection criteria adopted when, in a single maternal death notification, there were ICD-10 codes belonging to both direct and indirect obstetric cause groups. Similarly, it is necessary to clarify the procedure followed when the codes O98.5 and O99.5 appeared in the same maternal death notification. There is an example on page 12 of the document "International Guidelines for Certification and Classification (Coding) of COVID-19 as Cause of Death" . | Selection criteria  The study focused on the main cause of maternal death as published by DEIS Chile; secondary or underlying causes were not published and, thus, were excluded. This approach is consistent with DEIS Chile’s method of reporting the primary cause of death. For maternal deaths related to SARS-CoV-2, code O98.5 was assigned as the principal cause (see page 2, lines 149-153). |
|  |  |  |  |
| 10 | Reviewer 1 | c) The authors applied three statistical methods for time-series analysis: linear logarithmic regression (Joinpoint), interrupted time series (ITS), and autoregressive integrated moving average (ARIMA). Although the three methods together provide more information, it has resulted in an extended manuscript. A suggestion for the authors is to leverage the Joinpoint analysis to produce another manuscript, and in this one, to prioritize ITS and ARIMA analyses, thereby making the text more concise and focused on the objective of assessing the impact of the COVID-19 pandemic on general maternal mortality and by subgroups of causes. | To prioritize ITS and ARIMA analyses, the linear logarithmic regression analysis (Joinpoint), was omitted from the manuscript (see page2, lines 48-49, pages 2-3, lines 155-165 and page 4-6, lines 218-247). |
|  |  |  |  |
| 11 | Reviewer 1 | d) The statistical analyses are well described and appropriate. However, in Figure 2, it can be seen that, in some time series, the regression line for the pre-interruption period does not seem to fit the points so well. I wonder if the authors tested analyzing different sizes of time series (different temporal intervals) and/or statistical methods for ITS, to assess the robustness of the results. | Regarding Figure 2, we acknowledge that there are instances where the regression line for the pre-interruption period may not align perfectly with the data points. We agree that this observation warrants further investigation. To address this concern, we took the following steps: We began by using the Newey-West method to directly address autocorrelation. This robust approach adjusts standard errors and accounts for potential serial correlation in the time series data. Additionally, we explored the Prais-Winsten method. While it assumes an AR(1) dependence structure, it can be more efficient under certain conditions. Our goal was to balance robustness and efficiency in our analyses. We conducted sensitivity analyses to assess the robustness of our results. First, varying autocorrelation structures. Despite variations in the assumed autocorrelation structures (even beyond what was directly observed in the data), the overall patterns remained consistent. Second, performing alternative ITS methods. We applied segmented regression models and autoregressive integrated moving average (ARIMA) models. The results from these alternative methods are closely aligned with our main findings, reinforcing the stability of our conclusions. We’ve updated the manuscript to include a brief presentation of these sensitivity analyses in the appropriate section. In our discussion, we emphasize the need for caution when interpreting results based on annual data. Fine-grained analyses would enhance the robustness of our evidence. In summary, our original results are consistent even when considering different autocorrelation structures and statistical approaches. Then we conducted sensitivity analyses to assess the robustness of our results varying autocorrelation structures of the series (see page 3, lines 178-181). |
|  |  | [Turner, S.L., Karahalios, A., Forbes, A.B. et al. Comparison of six statistical methods for interrupted time series studies: empirical evaluation of 190 published series. BMC Med Res Methodol 21, 134 (2021). https://doi.org/10.1186/s12874-021-01306-w](https://doi.org/10.1186/s12874-021-01306-w) | We appreciate your kind contributions and the recommended bibliography. |
|  |  |  |  |
| 12 | Reviewer 1 | Results and Discussion: a) The results and discussion sections are well-written. However, I have a concern regarding the following interpretations in the discussion: |  |
|  |  | "Our findings suggest that SARS-CoV-2 predominantly impacts women with pre-existing conditions and comorbidities, such as diabetes and obesity." (Lines 382-383) | Thank you for the observation. We have modified the text according to your recommendation, including the following paragraph: Another hypothesis suggests that this novel coronavirus predominantly affects the MMR from indirect obstetric causes that are frequently associated with pre-existing conditions such as diabetes and obesity. However, the absence of detailed information on underlying causes hinders the differentiation between pregnant/postpartum with comorbidities or chronic diseases. To advance, further research is essential for a comprehensive understanding of these complex interactions (see page 12, lines 423-432). |
|  |  | "This implies that the increase in maternal mortality primarily occurred among women with pre-existing comorbidities." (Lines 412-413) | Thanks for the observation. The frase was eliminated from the text (see page 12, lines 464-466). |
|  |  | It is unclear how the authors arrived at this conclusion, given that the study's data were aggregated, and it probably was not possible to differentiate whether the pregnant/postpartum women who died from COVID-19 had comorbidities like diabetes, obesity, etc. COVID-19 can lead to maternal death, even in the absence of comorbidities. Furthermore, ARIMA and ITS analyses indicate an increase in maternal deaths mainly due to indirect infectious causes, while maternal deaths from indirect non-infectious causes remained within the forecasts for 2020 and 2021. | Thank you for your observation. We appreciate your feedback. Based on your input, we have adjusted our conclusions to more appropriately consider the nature of the data source and the results. As a result, any statements that implied a connection to individual people rather than aggregate data have been removed (see page 2, lines 64-68 and page 12, lines 464-466).  . |
|  |  |  |  |
| 13 | Reviewer 1 | b) It is recommended to comment on the coverage and quality of data; about the possibility of underreporting of maternal deaths in the country; the possibility that diagnoses from the group of indirect respiratory causes (pneumonia; acute respiratory distress syndrome) might have been cases of undiagnosed Covid-19. | Thanks for the observation. We include among the limitations the coverage and quality of data; and the possibility of underreporting or misclassification of maternal deaths in the country (see page 12, lines 447-454). |
|  |  |  |  |
| 14 | Reviewer 2 | The conclusion is a bit long perhaps general considerations should be moved to the discussion to keep a short conclusion with a crisp message on what was the hypothesis and what was found and the concordance of the 2 methods. | Thanks for the observation. We keep a short conclusion as recommended (see page 13, lines 471-480).  . |
| 15 | Reviewer 2 | The analysis is complex and has several components so the message is not so easy to wrap one's head around. | We removed the joint point to simplify the analysis of the results (see pages 2-3, lines 155-165 and page 4-6, lines 218-247) |

Kind regards,

Elard Koch

Corresponding author
